# Astrocytes expressing Vitamin D‐activating enzyme identify Parkinson’s disease

**DOI:** 10.1111/cns.13801

**Published:** 2022-02-15

**Authors:** Samanta Mazzetti, Michela Barichella, Federica Giampietro, Angelica Giana, Alessandra M Calogero, Alida Amadeo, Nicola Palazzi, Alessandro Comincini, Giorgio Giaccone, Manuela Bramerio, Serena Caronni, Viviana Cereda, Emanuele Cereda, Graziella Cappelletti, Chiara Rolando, Gianni Pezzoli

**Affiliations:** ^1^ Department of Biosciences Università degli Studi di Milano Milan Italy; ^2^ Fondazione Grigioni per il Morbo di Parkinson Milan Italy; ^3^ Parkinson Institute ASST “G.Pini‐CTO,” Milan Milan Italy; ^4^ Unit of Neuropathology and Neurology Fondazione IRCCS Istituto Neurologico Carlo Besta Milan Italy; ^5^ S. C. Divisione Oncologia Falck and S. C. Divisione Anatomia Patologica Ospedale Niguarda Ca' Granda Milan Italy; ^6^ Clinical Nutrition and Dietetics Unit Fondazione IRCCS Policlinico San Matteo Pavia Italy; ^7^ Center of Excellence on Neurodegenerative Diseases, Università degli Studi di Milano Milan Italy

**Keywords:** astrocytes, CYP27B1, Parkinson's disease, Vitamin D, α‐Synuclein oligomers

## Abstract

**Introduction:**

Astrocytes are involved in Parkinson's disease (PD) where they could contribute to α‐Synuclein pathology but also to neuroprotection via α‐Synuclein clearance. The molecular signature underlying their dual role is still elusive. Given that vitamin D has been recently suggested to be protective in neurodegeneration, the aim of our study was to investigate astrocyte and neuron vitamin D pathway alterations and their correlation with α‐Synuclein aggregates (ie, oligomers and fibrils) in human brain obtained from PD patients.

**Methods:**

The expression of vitamin D pathway components CYP27B1, CYP24A1, and VDR was examined in brains obtained from PD patients (Braak stage 6; *n* = 9) and control subjects (*n* = 4). We also exploited proximity ligation assay to identified toxic α‐Synuclein oligomers in human astrocytes.

**Results:**

We found that vitamin D‐activating enzyme CYP27B1 identified a subpopulation of astrocytes exclusively in PD patients. CYP27B1 positive astrocytes could display neuroprotective features as they sequester α‐Synuclein oligomers and are associated with Lewy body negative neurons.

**Conclusion:**

The presence of CYP27B1 astrocytes distinguishes PD patients and suggests their contribution to protect neurons and to ameliorate neuropathological traits.

## Graphical abstract

The vitamin D‐activating enzyme CYP27B1 identifies a subpopulation of astrocytes in PD patients. CYP27B1 positive astrocytes sequester α‐Synuclein oligomers and contact Lewy body negative neurons. In conclusion, the presence of these astrocytes distinguishes PD patients and suggests their contribution to protect neurons that could ameliorate neuropathological traits.

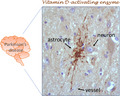

## 1 INTRODUCTION

Astrocytes are key players in Parkinson's disease (PD).^1^, ^2^, ^3^ α‐Synuclein misfolding and aggregation in neurons is the key hallmark of PD that associates with neuropathological deterioration.^4^ Astrocytes positively respond in PD as they can internalize and degrade α‐Synuclein aggregates thus protecting neurons.^1^, ^5^. On the contrary, the acquisition of inflammatory and altered metabolic properties in astrocytes results in detrimental effects and can potentially exacerbate the damage.^6^, ^7^ Therefore, identifying glial specific mechanisms that promote the acquisition of protective phenotype can be beneficial for treating PD.

The activation of vitamin D pathway has been linked to neuroprotection.^8^ Vitamin D_3_ (25‐hydroxyvitamin D [25(OH)D]) is present in the serum, and it needs to be activated by 25‐hydroxyvitamin D1‐α‐hydroxylase (CYP27B1) in order to exert its biological function.^9^ CYP27B1 and 1,25‐dihydroxyvitamin D receptor (VDR) are expressed by neurons and glial cells in *post*‐*mortem* human brain indicating that the vitamin D can be locally activated.^10^, ^11^ PD patients are usually characterized by lower 25(OH)D levels than controls^12^ and reduced serum 25(OH)D and polymorphisms in VDR have been associated with increased susceptibility to PD.^13^ Furthermore, lower serum concentrations have been observed in patients with the more severe symptoms including impaired cognitive functions.^14^, ^15^, ^16^, ^17^ Interestingly, vitamin D supplementation can slow Hoehn‐Yahr stage deterioration, particularly in patients carrying vitamin D receptor polymorphisms.^13^ In addition, vitamin D supplementation in PD patients has been recently suggested to play a protective role also in COVID‐19 symptoms.^18^

Despite the important role of vitamin D in PD, it is unknown whether the expression of the key signaling components is altered during the disease.^14^ We therefore aimed to analyze the distribution and localization of vitamin D‐activating and degrading enzymes CYP27B1 and CYP24A1 (25‐Hydroxyvitamin D‐24‐hydroxylase) and VDR in *post*‐*mortem* human brain (Braak stage 6) of PD patients. We observed that CYP27B1 was specifically increased in an astrocyte subpopulation of PD patients, exclusively in brain regions involved in the pathology. Furthermore, we investigated the involvement of CYP27B1 positive astrocytes in α‐Synuclein pathology and we correlated their expression with α‐Synuclein oligomers or Lewy bodies. These data all together could provide novel insights into vitamin D involvement in human astrocyte response during PD.

## 2 MATERIALS AND METHODS

### 2.1 Patients

The clinical diagnosis of PD was performed using the UK Brain Bank criteria^19^, ^20^ and confirmed by neuropathological analysis carried out by two experts (GG and MB) in agreement with the current BrainNet Europe Consortium guidelines.^21^

*Post*‐*mortem* human brains obtained from PD patients (*n* = 9; eight Braak stage 6 of α‐Synuclein pathology; one amigdala prevalence) and from control subjects (*n* = 4) clinically free from neurological diseases were used (Table 1; Nervous Tissues Bank of Milan). Written informed consent was obtained from all subjects in compliance with relevant laws and institutional guidelines and approved by the appropriate institutional committees.

**TABLE 1:** Demographic and clinical characteristics of the subjects included in the present study. All the patients were enrolled and followed during their disease by neurologists experienced in movement disorders at the Parkinson's Institute ASST G. Pini‐CTO of Milan

Group	Gender	Age at onset (years)	Age at death (years)	Disease duration (years)	Hoehn‐Yahr stage^a^	Dementia^b^
CTRL	M	/	71	/	/	No
CTRL	F	/	93	/	/	No
CTRL	F	/	82	/	/	No
CTRL	F	/	64	/	/	No
PD	M	62	80	18	4	No
PD	M	62	73	11	3	No
PD	M	59	87	28	3	No
PD	M	57	71	14	5	Yes
PD	M	57	75	18	3	No
PD	F	53	91	38	5	Yes
PD	F	59	79	20	4	No
PD	F	55	79	24	4	No
PD	F	43	72	29	5	Yes

Note

Abbreviations: CTRL, Control; F, female; M, male; PD, Parkinson's disease.

^a^ As assessed 1 year before death;

^b^ As defined by UPDRS‐Part I, item 1 score ≥ 2 and also mini‐mental state examination (MMSE) if necessary.

Brains were fixed in 10% buffered formalin for at least 1 month at 20°C. After dehydration and clearing steps, selected areas were paraffin embedded and 5 μm thick frontal cortex, mesencephalic sections, containing *substantia nigra*, pons, and bulb were cut and processed for the following analysis.

### 2.2 Reagents

Primary and secondary antibodies and kit are summarized in Table 2.

**TABLE 2:** Primary and secondary antibodies and kits used in this study

Antigen	Code	Host	Dilution
Primary antibodies			
25‐hydroxyvitamin D1‐α‐hydroxylase (CYP27B1)	ABN182 Merck	Rabbit	1:50 (IHC) 1:1500 (IF)
25‐Hydroxyvitamin D − 24‐hydroxylase (CYP24A1)	HPA022261 Merck	Rabbit	1:100
Apolipoprotein E (ApoE)	AB947 Merck	Goat	1:300
C3D Complement (C3)	A0063 Dako	Rabbit	1:100
Glial Fibrillary Acidic Protein (GFAP)	Z0334 Dako	Rabbit	1:300
Lysosome‐associate Membrane Protein 1 (LAMP1)	L1418 Sigma	Rabbit	1:100
S100β	Z0311 Dako	Rabbit	1:250
S100β	287006 Synaptic Systems	Chicken	1:1000
Tyrosine Hydroxylase (TH)	PA − 18372 Thermo Fisher	Goat	1:200
Vitamin D Receptor (VDR)	Sc − 13133 clone D − 6 Santa Cruz	Mouse	1:50
α‐Synuclein	Ab27766 clone LB509 Abcam	Mouse	1:500
α‐Synuclein	S3062 Merck	Rabbit	1:2000

Fluorochrome/Enzyme Antibody	Code	Host	Dilution
Secondary antibodies			
Alexa Fluor^®^ 488 anti‐rabbit	A21206 Thermo Fisher	Donkey	1:200
Alexa Fluor^®^ 568 anti‐goat	A11057 Thermo Fisher	Donkey	1:200
Alexa Fluor^®^ 647 anti‐rabbit	A32795 Thermo Fisher	Donkey	1:200
Alexa Fluor^®^ 647 anti‐goat	Jackson ImmunoResearch	Donkey	1:400
Cy3 anti‐chicken	Jackson ImmunoResearch	Donkey	1:400
HRP anti‐rabbit	Jackson ImmunoResearch	Donkey	1:5000
EnVision System‐HRP Labeled polymer anti‐mouse	K4001 Dako Omnis	Goat	1:1
EnVision System‐HRP Labeled polymer anti‐rabbit	K4003 Dako Omnis	Goat	1:1
ImmPRESS™‐AP anti‐ rabbit	MP − 5401Vector	Horse	1:1
Commercial assay			
Duolink^®^ in situ probe marker MINUS	DUO920101KT Merck	‐	*
Duolink^®^ in situ probe marker PLUS	DUO920091KT Merck	‐	*
Duolink^®^ In Situ Detection Reagents Red	DUO92008 Merck	‐	*
EnVision FLEX DAB + SubstrateChromogen System	K3468 Dako	‐	*
EnVision™ Flex Target retrieval solution high pH	K8004 Dako	‐	*
Fast Blue B salt	F3378‐1G Merck	‐	1 mg/ml
Hoescht 33342	62249 Thermo Fisher	‐	1:5000
TSA^®^ Plus Fluoresceine	NEL741001KT Akoya Biosciences	‐	1:50

* Used as indicated by the manufacture instruction.

### 2.3 Immunohistochemistry

Antigen retrieval (80% formic acid for 20 min at room temperature, RT) was necessary only for VDR staining. Samples were incubated for 20 min with 3% H_2_O_2_ followed by 1% BSA diluted in 0.01 M phosphate saline buffer (PBS) containing 0.1% Triton X‐100 (PBS‐T). Primary antibody (anti‐VDR, anti‐CYP27B1, anti‐Glial fibrillary acidic protein, GFAP) in 1% BSA diluted in PBS‐T was incubated overnight at RT. Antigen‐antibody bound was visualized using goat anti‐mouse (for VDR) or anti‐rabbit (for CYP27B1 and GFAP) secondary antibody (1 h, RT) and with 3,3’‐Diaminobenzidine as chromogen (DAB kit).

In order to associate Lewy body pathology to CYP27B1 staining, we performed a double immunoenzymatic procedure using anti α‐Synuclein (1:500, LB509). The samples were also pretreated with 20% acetic acid for 20 min to inactivate endogenous alkaline phosphatase. The secondary antibodies used were goat anti‐mouse conjugated with HRP and rabbit conjugated with alkaline phosphatase. Then, a substrate solution of 0.1 M Tris‐HCl (pH 9.2–9.4), 1‐Naphthyl phosphate disodium salt (1mg/ml), and Fast Blue B salt (1mg/ml) was used to develop the CYP27B1 staining and DAB incubation to visualize α‐Synuclein.

### 2.4 Immunofluorescence and confocal analysis

To detect CYP27B1, we used tyramide signal amplification system. The sections were incubated with: (i) CYP27B1 (1:1500) in 1% BSA and 0.3% Triton X‐100 diluted in TN (0.1 M Tris‐HCl, 0.15 M NaCl) overnight at RT; (ii) HRP‐conjugated secondary antibodies (donkey anti‐rabbit, 1:5000) in TN for 2 h at RT; (iii) FITC‐labeled tyramide (1:50) in Amplification Diluent for 2 min at RT. Classical immunofluorescence was performed with different primary antibodies (anti‐GFAP, 1:300 or S100β 1:250, and Apolipoprotein E 1:300; or C3D Complement 1:100; or Tyrosine Hydroxylase 1:200; or LAMP1 1:100 overnight at RT) followed by highly pre‐adsorbed secondary antibodies (2 h). A control was performed using CYP27B1 diluted 1:1500 revealed with Alexa Fluor 488 donkey anti‐rabbit and gave no signal. Proximity ligation assay was used for α‐Synuclein oligomers using red amplification reagent, as previously described.^22^ In order to visualize total α‐Synuclein, Alexa Fluor 647 donkey anti‐rabbit (1:200) was added during polymerase step. Hoescht 33342 (1:5000 for 10 min) was used for nuclei counterstaining. Samples were mounted using Mowiol‐DABCO and examined with Nikon spinning disk confocal microscope, equipped with CSI–W1 confocal scanner unit.

### 2.5 Quantification and statistical analysis

All quantifications were independently performed using ImageJ (cell counter) by two different operators (FG, SM, or CR). Statistical comparisons were conducted by Mann‐Whitney and one‐way ANOVA tests. Statistical significance was assessed using GraphPad Prism software. Significance was established at *p* < 0.05.

### 2.6 Data availability

The datasets used and analyzed during the current study are available from the corresponding author upon reasonable request.

## 3 RESULTS

### 3.1 Vitamin D‐activating enzyme is overexpressed in astrocytes of PD patients

We tested whether vitamin D signaling components are altered in PD. In *substantia nigra*, VDR localized in the nucleus and cytoplasm of neuromelanin containing neurons with no differences between controls and PD patients (Figure S1 A‐C). Besides, vitamin D degrading enzyme CYP24A1 was not altered in PD and was mainly expressed in neurons as in controls (Figure S1 D‐E″).^23^

**FIGURE 1:**
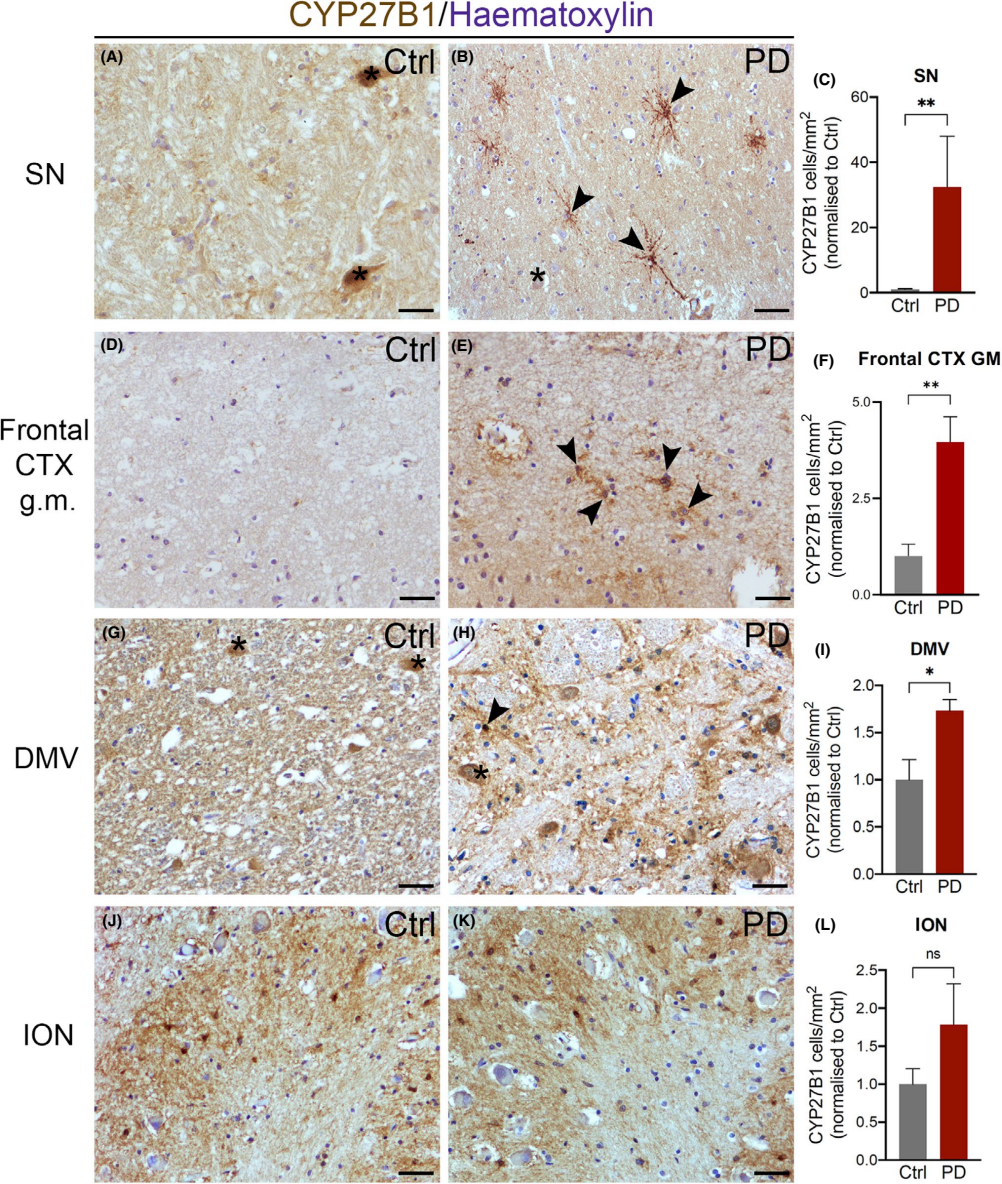
CYP27B1 distribution in *post*‐*mortem* human brain. In *substantia nigra* (SN; A and B) and in dorsal motor nucleus of vagus (DMV; G and H), CYP27B1 staining is mainly localized in neuronal cell bodies and in neuropil of control subjects (Ctrl; A and G), while in PD patients, CYP27B1 intense staining is visible in astrocytes with a morphology resembling varicose projection astrocytes (PD; B and H; black arrowheads). In layer I of frontal cortex gray matter (Frontal CTX g.m., D and E), a low staining is detectable in controls (D), while in PD patients CYP27B1 astrocytes are well visible (E). In inferior olivary nucleus (ION; J and K), CYP27B1 staining is present in cell bodies of astrocytes in both control and PD samples. Nuclei are stained with hematoxylin. Black asterisks: dopaminergic neurons containing neuromelanin. Scale bar, 40 μm. Graphs show the density of CYP27B1 positive astrocytes in controls and PD patients in SN (C, 22 *vs* 694), Frontal Cortex GM (F, 245 *vs* 2307), DMV (I, 428 *vs* 793), and ION (L, 374 *vs* 461). Data in graphs are reported as mean ± standard error; Mann‐Whitney test (* *p* < 0.05, ** *p* < 0.01)

**FIGURE 2:**
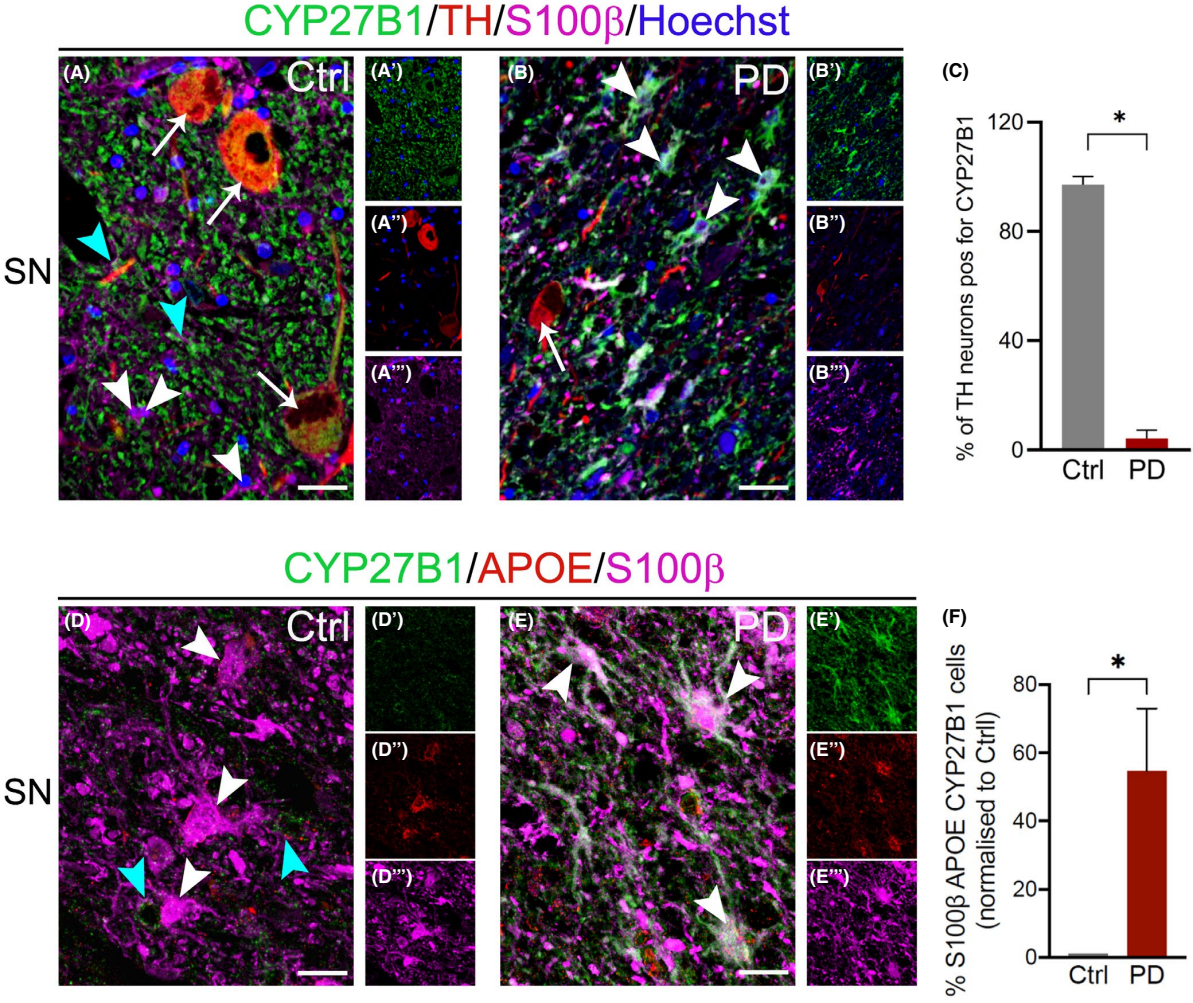
Confocal analysis of CYP27B1 positive cells in *substantia nigra* of control subjects and PD patients. CYP27B1 staining is intense in TH positive neurons (A, B; white arrows) in controls (A‐A’’) but not in PD (B‐B’’). On the contrary, in S100β positive astrocytes (A, B; white arrowheads), CYP27B1 staining is intense in PD (B, B’, B’’’), while it is restricted to astrocyte processes (A, A’, A’’’; cyan arrowheads) in controls. Nuclei are counterstained with Hoechst. Scale bar, 30 μm. Graph shows the percentage of TH neurons positive for CYP27B1 (113 neurons analyzed for controls, *n* = 3, and 214 for PD, *n* = 6). CYP27B1 staining is intense and diffuse in cell bodies and processes of ApoE and S100β positive astrocytes (white arrowheads) in PD samples (E‐E’’’), while it is visible in astrocytic end‐feet of controls (D‐D’’’; cyan arrowheads). Scale bar, 15 μm. Graph (F) shows the percentage of astrocytes positive for S100β, APOE, and CYP27B1 (137 astrocytes analyzed for controls, *n* = 3, and 118 for PD, *n* = 3). Data in graphs (C, F) are reported as mean ± standard error; Mann‐Whitney test (* *p* < 0.05)

**FIGURE 3:**
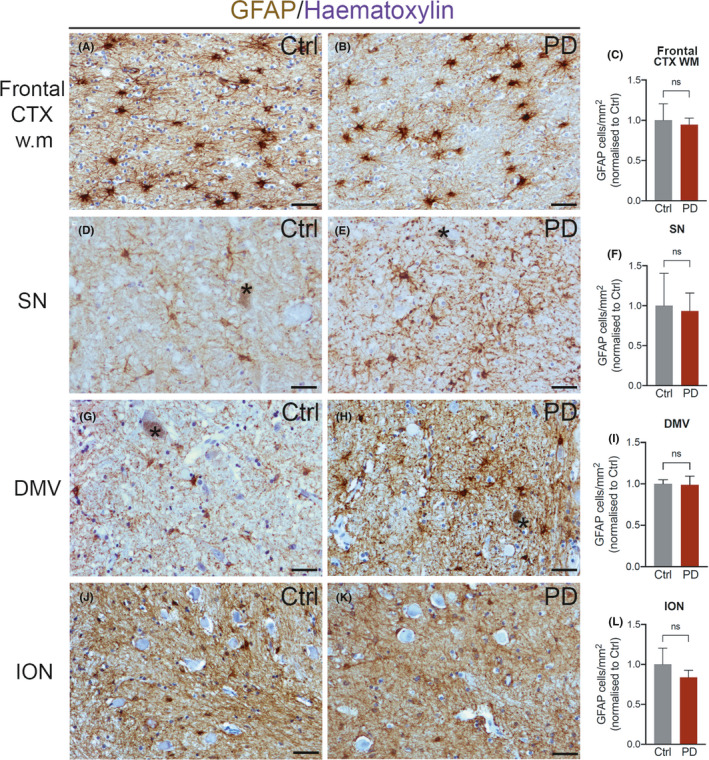
GFAP distribution in *post‐mortem* human brain. In frontal cortex white matter (Frontal CTX w.m.; A and B), *substantia nigra* (SN; D and E), dorsal motor nucleus of vagus (DMV; G and H), and inferior olivary nucleus (ION; J and K), GFAP staining is localized in human astrocytes in control and PD samples. Nuclei are stained with hematoxylin. Black asterisks: dopaminergic neurons containing neuromelanin. Scale bar, 40 μm. Graphs show the density of GFAP positive astrocytes in controls and PD patients Frontal Cortex WM (C, 1527 vs 3363), SN (F, 776 vs 897), DMV (I, 850 vs 1029), and ION (L, 1281 vs 1895). Data in graphs are reported as mean ± standard error; Mann‐Whitney test (n.s.)

In *substantia nigra*, CYP27B1 was mainly present in dopaminergic neurons as revealed by the presence of neuromelanin (Figure 1 A) and the staining with TH (Figure 2 A‐A‴). The percentage of dopaminergic neurons positive for CYP27B was significantly decreased in PD (Figure 2 B‐C) compared to control subjects (Figure 2 A‐A‴). This result could suggest that a less activation of vitamin D is implicated in the disease. Furthermore, in PD, CYP27B1 labeled a subpopulation of parenchymal astrocytes with long processes and varicosities that resembled the varicose projection astrocytes exclusively found in human brain (Figure 1 B,C, Figure 2 E‐E‴ and Figure 5 A).^24^, ^25^ CYP27B1 positive astrocytes increased also in other areas involved in the pathology, including the frontal cortex (gray matter, Figure 1 D‐F; white matter, Figure 7 A,B,D) and the dorsal motor nucleus of vagus (Figure 1 G‐I), while no differences were observed in the inferior olivary nucleus that is instead spared by the disease (Figure 1 J‐L). We confirmed that CYP27B1 positive cells in PD are astrocytes expressing the astrocytic markers S100β, Apolipoprotein E, and GFAP (Figure 2 D‐F; Figure S2 A‐B‴). Interestingly, the increase in CYP27B1 positive astrocytes was not the result of a general expansion of astrocytes in PD, as the number of GFAP positive cells was unchanged between control subjects and PD patients (Figure 3 A‐L), but it could be the result of a neuroprotective response. In summary, CYP27B1 overexpressing astrocytes are enriched in PD patients.

### 3.2 CYP27B1 positive astrocytes and α‐Synuclein pathology

**FIGURE 4:**
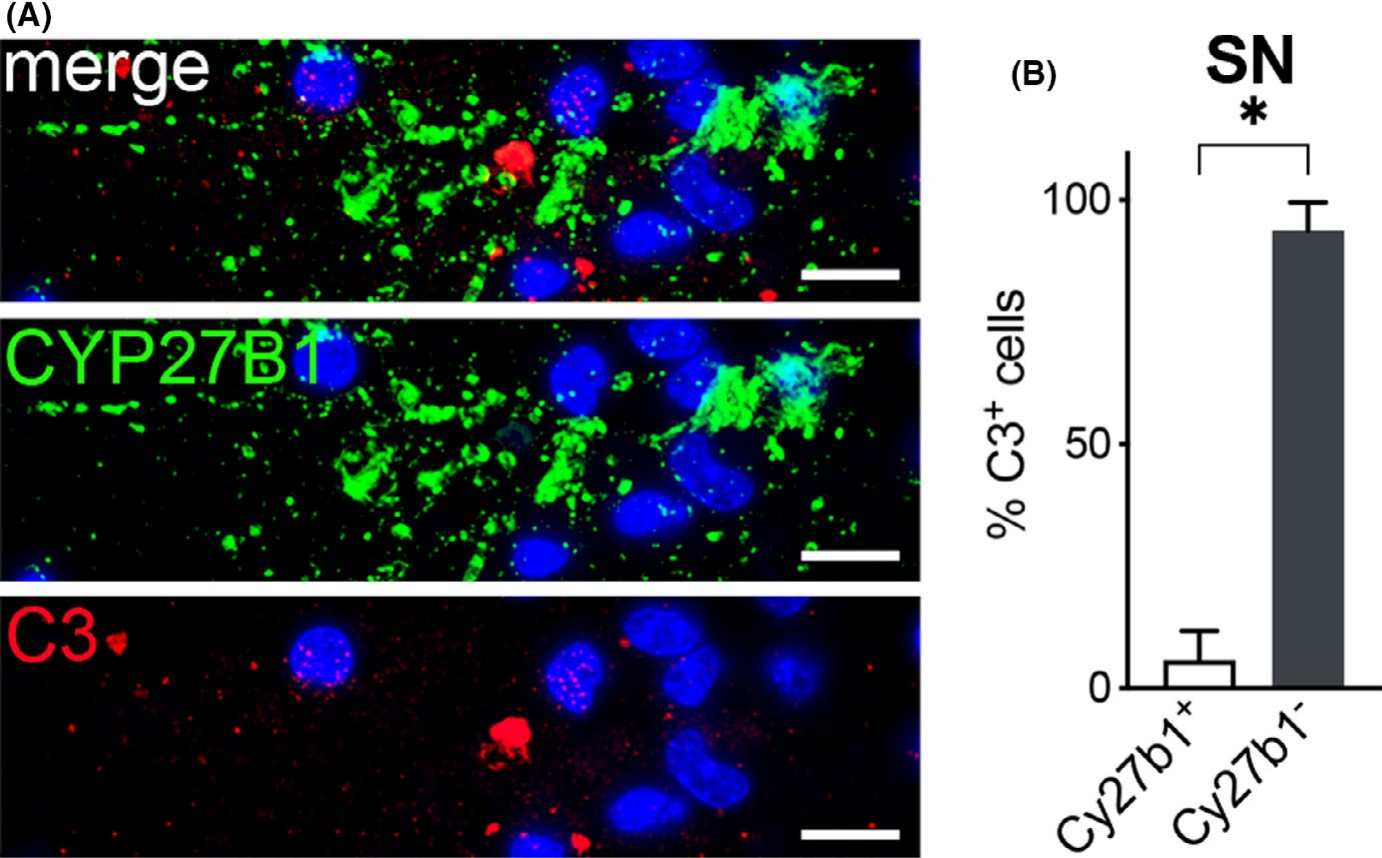
CYP27B1 positive astrocytes do not display neurotoxic signature. CYP27B1 positive astrocyte (green) and C3 (red) staining in PD samples (A). Nuclei are counterstained with Hoechst (blue). Scale bar, 10 μm. Graph (B) shows the percentage of C3 cells containing CYP27B1 (200 cells analyzed, *n* = 4 patients). Data in graph are reported as mean ± standard error; Mann‐Whitney test (* *p* < 0.05)

We tested whether CYP27B1 positive astrocyte accumulation is beneficial or detrimental for PD neuropathology. During PD, astrocytes can acquire a neurotoxic phenotype that exacerbate neuronal damage.^6^ To determine whether CYP27B1 positive astrocytes have a neurotoxic signature, we analyzed the expression of complement component 3 (C3).^6^ We found that among all C3 positive cells in PD *substantia nigra*, only 4% were CYP27B1, thus indicating that they did not acquire a neurotoxic state (Figure 4 A,B).

**FIGURE 5:**
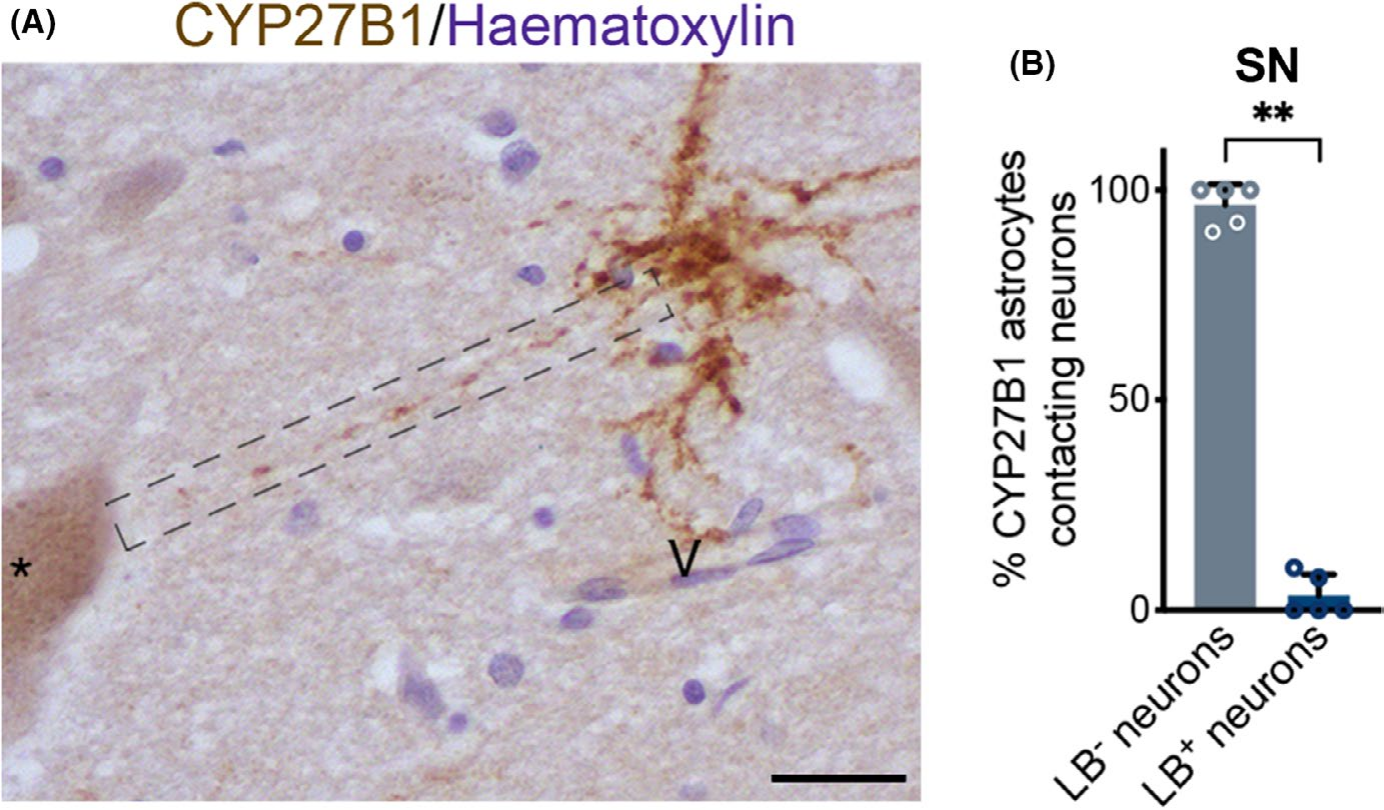
Correlation between CYP27B1 astrocytes and Lewy body pathology. CYP27B1 positive astrocyte characterized by a long varicose process (dashed rectangle) contacting a dopaminergic neuron containing neuromelanin (black asterisk) and a blood vessel (V) in PD sample (A). Nuclei are counterstained with hematoxylin. Scale bar, 30 μm. Graph (B) shows that most neurons contacted by CYP27B1 positive astrocytes do not contain Lewy bodies (530 neurons analyzed, n = 5 patients). Data in graph are reported as mean ± standard error; Mann‐Whitney test (** *p* < 0.01)

Next, we investigated the contribution of CYP27B1 positive astrocytes in α‐Synuclein pathology. Braak stage 6 dopaminergic neurons accumulate Lewy bodies in their cytoplasm.^26^, ^27^ We examined the relationship between dopaminergic neurons and CYP27B1 positive astrocytes in the *substantia nigra* (Figure 5 A). Interestingly, the majority of CYP27B1 positive astrocytes were in direct contact with neurons that did not contain Lewy bodies (Figure 5 B). Consistently with this data, we did not observe CYP27B1 positive astrocytes in *locus coeruleus*, where almost all the neurons we analyzed contained Lewy bodies (Figure S3 A‐D‴).

**FIGURE 6:**
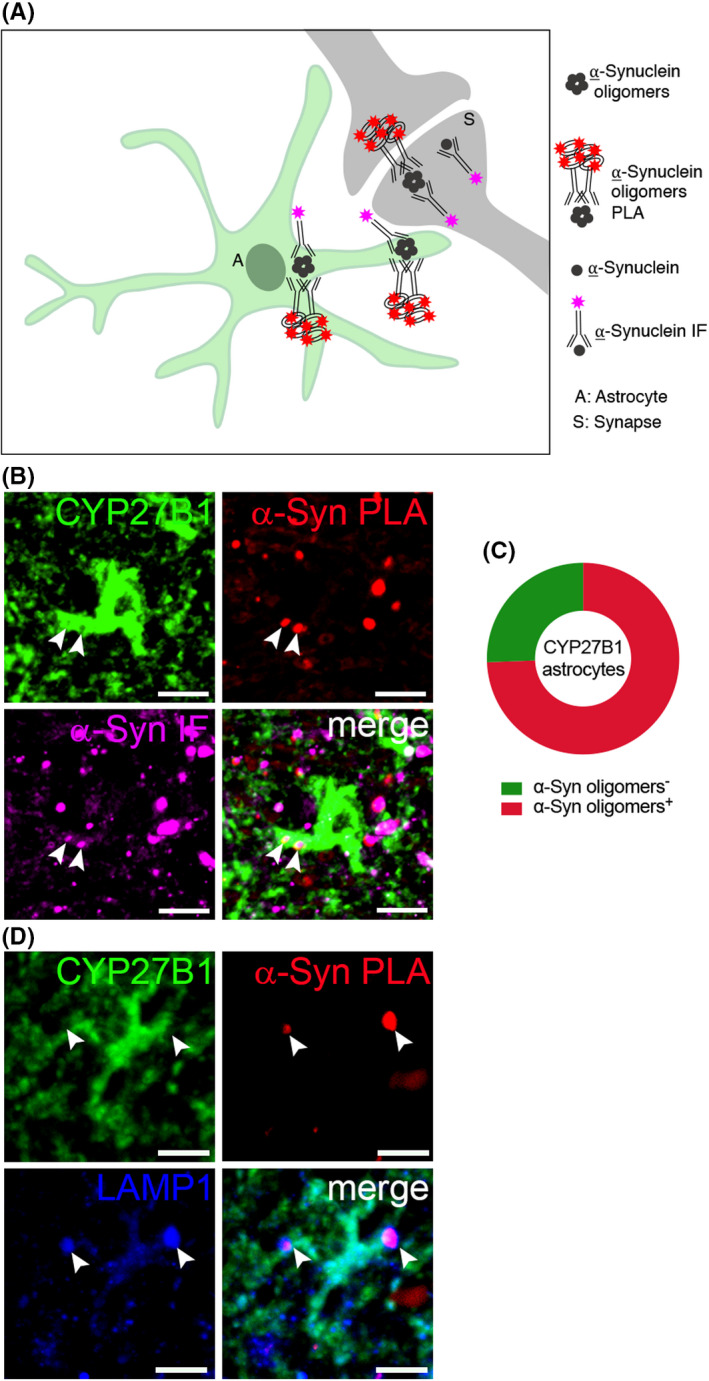
CYP27B1 astrocytes contain α‐Synuclein oligomers. (A) Scheme representing the experimental procedure to visualize the relationship between α‐Synuclein oligomers (proximity ligation assay, PLA) and total α‐Synuclein (immunofluorescence, IF) with CYP27B1 positive astrocyte. (B) CYP27B1 positive astrocytes (green) containing total α‐Synuclein staining (purple) and α‐Synuclein oligomers (red) in PD samples Scale bar, 25 μm. Graph (C) shows that 74.4% of CYP27B1 positive astrocytes (*n* = 43) bears α‐Synuclein oligomers. (D) CYP27B1 positive astrocytes (green) containing vesicles positive for both α‐Synuclein oligomers (red) and LAMP1 (blue). Scale bar, 40 μm

We therefore hypothesized that CYP27B1 positive astrocytes can be directly linked to α‐Synuclein uptake and clearance. We performed proximity ligation assay to detect α‐Synuclein oligomers *in vivo* and found that 74.4% of CYP27B1 positive astrocytes were able to uptake the toxic α‐Synuclein aggregates (Figure 6 A‐C and S4 A‐B‴). Then, we used LAMP1, a marker for degradative autophagy‐lysosomal organelles,^28^ in order to discover whether autophagy could be the mechanism involved in α‐Synuclein clearance in CYP27B1 positive astrocytes. Notably, we found that α‐Synuclein oligomers that are detected inside CYP27B1 positive astrocytes colocalize with LAMP1‐positive vesicles (Figure 6 D and S4 C‐D‴), thus suggesting the involvement of an autophagy pathway.

**FIGURE 7:**
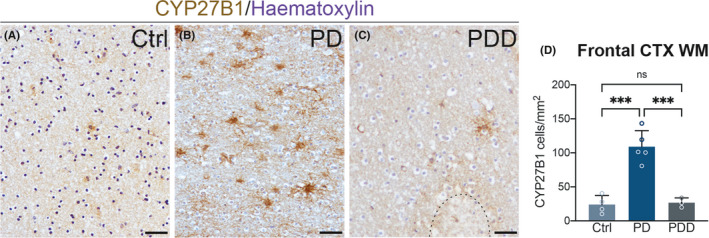
Comparison of the CYP27B1 positive astrocytes distribution in frontal cortex (white matter) between control (A), PD (B), and PD dementia (C) samples. In (C) is visible a white matter lesion (black outline). Graph (D) shows the number of CYP27B1 astrocytes normalized on area unit (807 astrocytes analyzed for controls, *n* = 4; 4594 for PD, *n* = 5, and 647 for PD dementia, *n* = 3). Nuclei counterstained with hematoxylin (blue). Scale bar, 40 μm. Data in the graph are mean ± standard error; one‐way ANOVA with Tukey's multiple comparison test (*** *p* < 0.001)

Finally, we examined whether the increase in CYP27B1 positive astrocytes correlated with clinical aspects of PD patients (Table 1). We observed that CYP27B1 expression in the frontal cortex white matter was significantly increased in patients with Lewy body pathology, but without white matter alteration or dementia (Figure 7 A,B,D). Conversely, PD patients with white matter alterations and concomitant dementia had significantly fewer CYP27B1 positive astrocytes in the cortex (Figure 7 C,D), despite the total GFAP positive cells were unchanged (Figure 3 C).

## 4 DISCUSSION

Our study unravels for the first time the alterations of vitamin D pathway in human brain of PD patients. While CYP24A1 and VDR did not display different expression between patients and controls, we showed that CYP27B1 increased in a subpopulation of astrocytes with neuroprotective features exclusively in brain areas involved in PD pathology (dorsal motor nucleus of vagus, *substantia nigra* and frontal cortex). Moreover, we found that CYP27B1 positive astrocytes are involved in an autophagy mediated‐α‐Synuclein uptake. These findings suggest an unprecedented role for CYP27B1 positive astrocytes in the pathology of PD.

Reactive astrocytes heavily contribute to neurodegeneration, and recent evidence suggests that neurotoxic astrocytes accumulate also in the *substantia nigra* of PD patients.^6^ The exact mechanism that drives astrocytes into detrimental alterations (eg, the secretion of neurotoxins) is unknown. Vitamin D prevents these astrocytes alterations in PD mouse model,^29^ while vitamin D signaling is unknown in PD patients. We found that activation of vitamin D pathway might be a novel mechanism to prevent the neurotoxic switch of astrocytes. Indeed, CYP27B1 positive astrocytes did not upregulate C3, thus protecting neurons from the complement component‐driven synapse degeneration.

Recent evidence suggests that astrocytes participate in α‐Synuclein oligomer clearance *in vitro* in primary cultures and patient‐derived iPSC model.^2^, ^5^, ^30^ Starting from these previous papers on cellular model, showing that α‐Synuclein fibrils co‐localized with LAMP1, a marker for degradative autophagy‐lysosomal organelles,^28^ we looked for the involvement of the autophagy pathway in α‐Synuclein clearance occurring in our samples. The strategy of staining with LAMP1 allowed us to highlight the involvement of autophagy in α‐Synuclein clearance that occurs in the complexity and uniqueness of the human brain. Hence, we show for the first time that an astrocyte subpopulation that is positive for CYP27B1 can internalize α‐Synuclein oligomers through autophagy in PD patients. Interestingly, these cells morphologically resemble varicose projection astrocytes that are known to be fast conduit in neuron‐vascular unit and are characterized by high speed calcium waves.^25^ In addition, the overexpression of wild‐type or familial mutant α‐Synuclein triggers calcium homeostasis alterations.^31^ Given the key role of vitamin D in calcium buffering,^32^ we speculate that the increase of CYP27B1 in PD astrocytes could be a positive strategy to counteract the calcium alterations. In line with that, CYP27B1 positive astrocytes are exclusively in contact with dopaminergic neurons without Lewy bodies suggesting their role in α‐Synuclein oligomers clearance and osmoregulatory function.

Vitamin D deficiency may lead to cognitive impairments.^14^, ^15^, ^16^, ^17^ Vitamin D supplementation can be beneficial in slowing down PD progression, but the underlying mechanisms are still unknown.^13^, ^33^ We found that the brains of PD patients without dementia have a threefold higher content of CYP27B1 positive astrocytes in the frontal cortex and do not have white matter degeneration, thus suggesting that vitamin D could exert its neuroprotective role through astrocytes.^11^ Although it would be appropriate to assess serum 25(OH)D and start supplementation to prevent PD or to slow its progression, the exact mechanisms underlying aberrant vitamin D pathways still need to be established. Considering that PD patients are usually characterized by low serum 25(OH)D,^12^ data in the different study groups presented low heterogeneity and VDR and CYP24A1 are similarly expressed in brains of PD patients and controls, a noteworthy question raised by our findings is how CYP27B1 can prevent neurotoxic alterations and promote α‐Synuclein uptake by astrocytes? A limitation of our study is that we could not assess 25(OH)D levels and genetic status of the analyzed patients, and thus, we cannot exclude the potential contribution neither of concomitant vitamin D deficiency nor of polymorphisms in VDR.^13^ Further studies are warranted to clarify the complex relationship between vitamin D activation in astrocytes and PD.

Finally, our neuropathological investigation links for the first time vitamin D to the clearance of α‐Synuclein aggregates and demonstrates that the presence of CYP27B1 positive astrocytes distinguishes PD patients.

## CONFLICT OF INTEREST

The authors reported no conflict of interest.

## AUTHOR CONTRIBUTIONS

Samanta Mazzetti, Chiara Rolando, Federica Giampietro, Michela Barichella, and Graziella Cappelletti contributed equally to the original research idea conception, study design, literature search, data collection, data analysis and interpretation, figure and table formatting, and writing of the manuscript. Angelica Giana, Alessandra M Calogero, Giorgio Giaccone, Viviana Cereda, and Emanuele Cereda contributed to data collection and analysis and assisted in the writing of the text. Alida Amadeo, Nicola Palazzi, Alessandro Comincini, Manuela Bramerio, and Serena Caronni contributed to data collection. Graziella Cappelletti and Gianni Pezzoli supervised the study and assisted in the writing of the text. All authors provided intellectual content and critical review of the manuscript.

## ETHICS STATEMENT

The study procedures were in accordance with the principles outlined in the Declaration of Helsinki and approved by the Ethics Committee of University of Milan (protocol code 66/21, 15.06.2021).

## Supporting information

Fig S1‐S4

## Data Availability

The dataset of this research is deposited in the official computer archive of the Cappelletti's laboratory (Department of Biosciences, University of Milan), and it is available upon request.
