# Supplementary material for: Astrocytes expressing Vitamin D‐activating enzyme identify Parkinson’s disease
Source: CNS Neurosci Ther. 2022 Feb 15;28(5):703–13. doi: 10.1111/cns.13801 (PMC8981451; doi:10.1111/cns.13801)
Supplement: Supplementary file 1 — Fig S1‐S4 [file CNS-28-703-s001.pdf]

SUPPORTING INFORMATION

Supplementary material

Immunofluorescence and confocal analysis

Antigen retrieval with EnVision™ Flex Target retrieval solution high pH (50x) was necessary for TH staining. After the blocking solution (1% BSA in PBS-T), a mix of primary antibodies (anti-CYP24A1, S100β and anti-Tyrosine Hydroxylase) was incubated overnight at RT on mesencephalic sections, containing *substantia nigra*. Samples then were treated for 2 hours at RT with a mix of secondary antibodies (Alexa Fluor 488 donkey anti-rabbit, Cy3 donkey anti-chicken and Alexa Fluor 647 donkey anti-goat). Hoescht 33342 (1:5000 for 10 minutes) was used for nuclei counterstaining. Finally, samples were mounted using Mowiol+DABCO and examined with A1 Nikon confocal microscope.

Supplementary figures

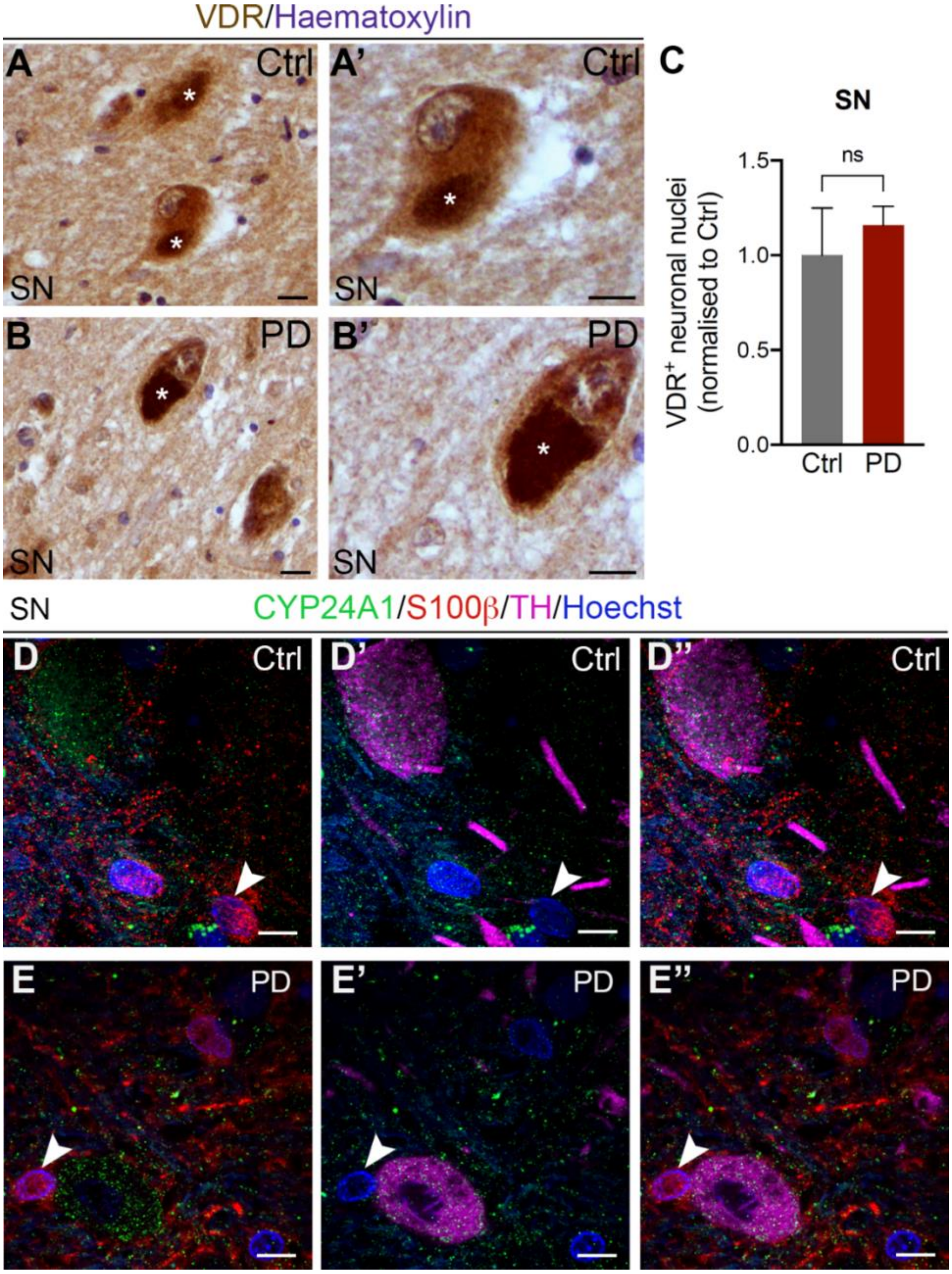

FIGURE S1 Distribution of VDR and CYP24A1 in substantia nigra of PD patients. (**A-C**) VDR staining is localized in cytoplasm and in nuclei of dopaminergic neurons both in control (**A-A'**) and PD (**B-B'**) samples. Nuclei are stained with haematoxylin. White asterisks: dopaminergic neurons containing neuromelanin. Graph **(C)** represents the ratio between VDR positive nuclei and total dopaminergic neurons present in the samples analysed. Data in graphs are reported as mean ± standard error and statistical comparisons were made using Mann-Whitney test. (**D-E''**) Confocal analysis of CYP24A1 (green), TH (purple) and S100β (red). CYP24A1 staining is mainly localized in cytoplasm of TH positive dopaminergic neurons in both control (D-D'') and PD samples (E-E''). In the majority of astrocytes, CYP24A1 is not present (arrowheads). (**A-B'**, **D-E"**) Scale bar, 20μm.

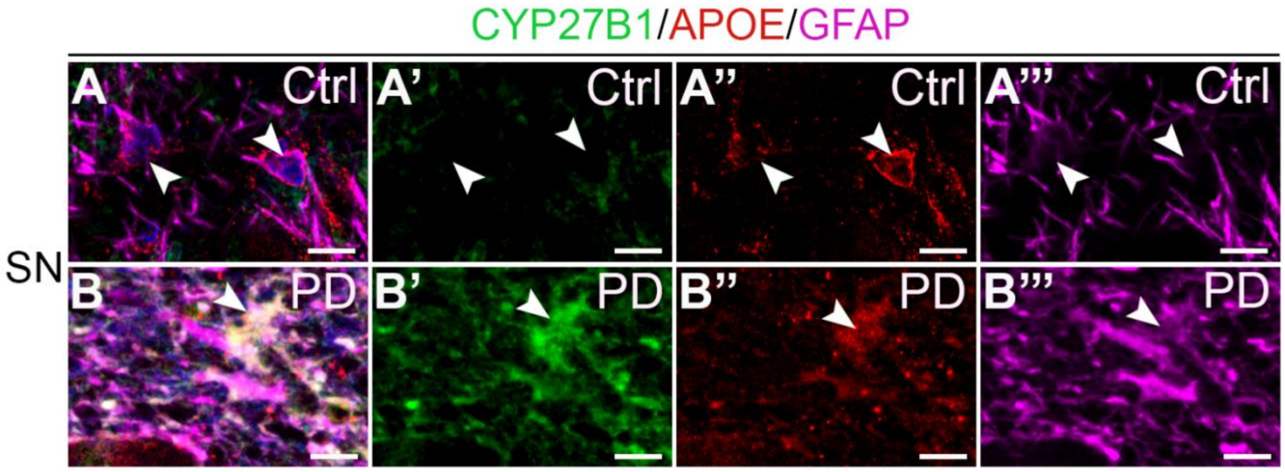

FIGURE S2 Confocal analysis of CYP27B1 (green), ApoE (red) and GFAP (purple) in *substantia nigra* of control (**A-A’’’**) and PD patients (**B-B’’’**). Nuclei are stained with Hoechst (blue). Scale bar, 15 μm.

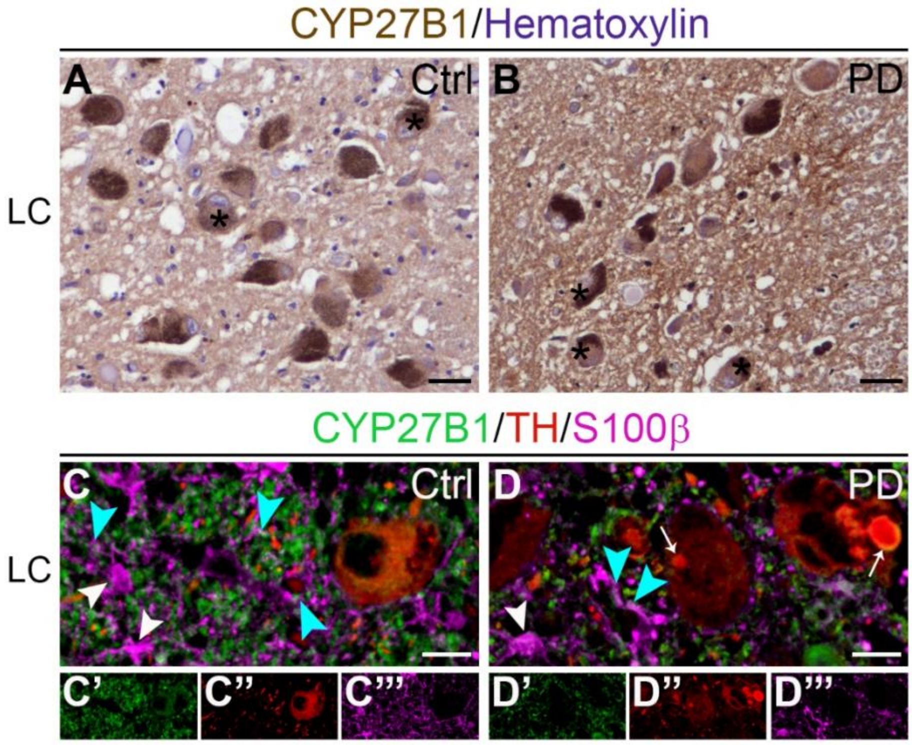

FIGURE S3 CYP27B1 distribution in *locus coeruleus* of *post-mortem* human brain. CYP27B1 is mainly distributed in neuronal cell bodies of control subjects (**A**) while it is abundant in neuropils of PD samples (**B**). Nuclei are stained with haematoxylin. Black asterisks: neurons containing neuromelanin. CYP27B1 staining is localized in TH positive neurons in controls (**C**-**C''**) but not in dopaminergic neurons containing Lewy bodies in PD (white arrows; **D**-**D''**). In S100β positive astrocytes CYP27B1 does not stain the cell bodies (white arrowheads) but only the astrocytic end-feet (cyan arrowheads) both in control (**C**, **C'**, **C''**) and in PD samples (**D**, **D'**, **D'''**). Scale bar, A, B 40 µm; C, D 30 µm.

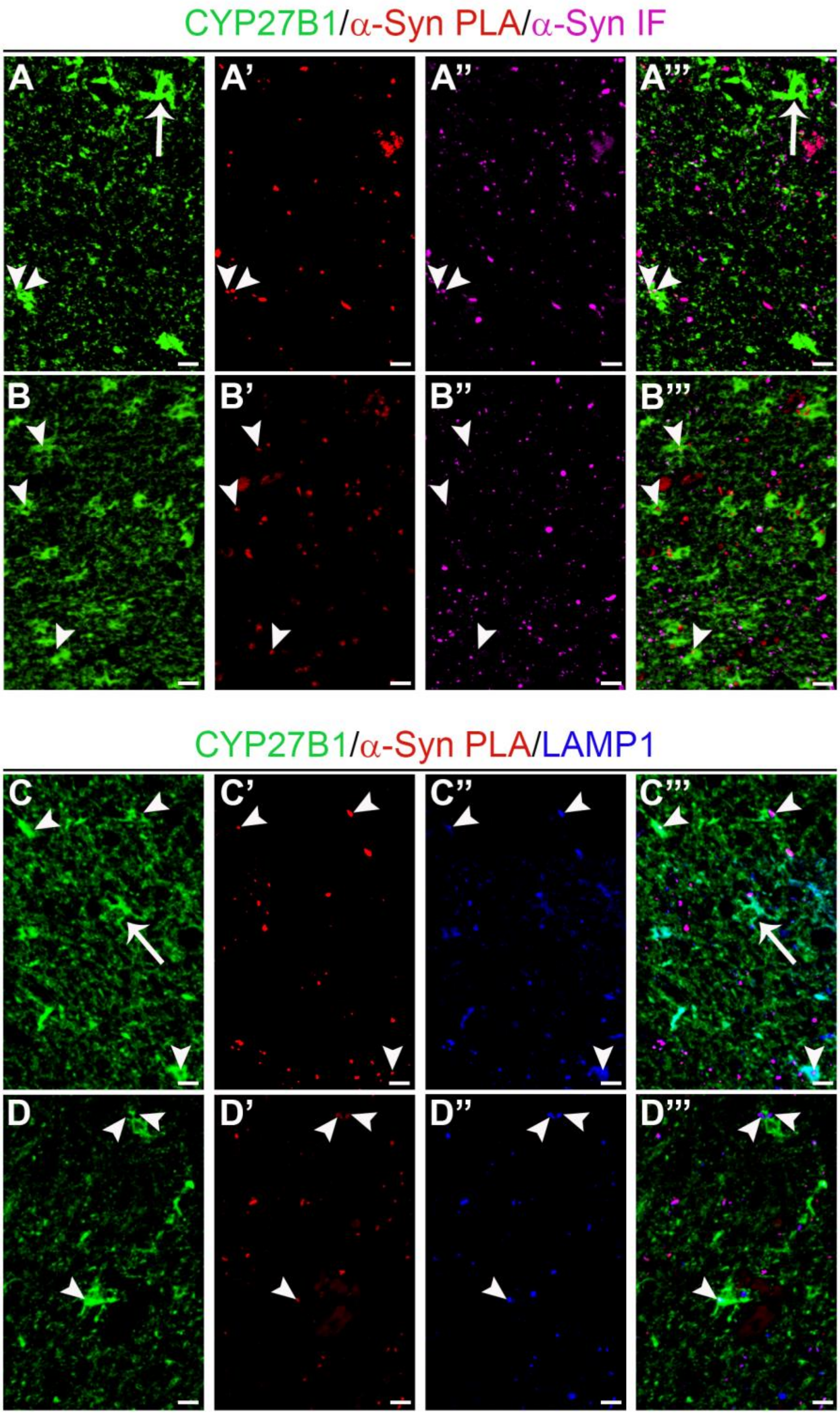

FIGURE S4 CYP27B1 astrocytes contain α-Synuclein oligomers. α-Synuclein oligomers (red, proximity ligation assay, PLA) are visualized in CYP27B1 positive astrocytes (green) in PD samples (arrowhead; **A-B'''**). Total α-Synuclein staining (purple, immunofluorescence, IF) is present also in the synaptic terminal outside glial cells. Furthermore, CYP27B1 positive astrocytes (green) containing vesicles positive for both α-Synuclein oligomers (red) and LAMP1 (blue) are present in PD samples (arrowhead; **C-D'''**). Scale bar, 20 μm. White arrows indicate astrocytes magnified in figure 6.
